# One-Step Electrochemical Fabrication of Reduced Graphene Oxide/Gold Nanoparticles Nanocomposite-Modified Electrode for Simultaneous Detection of Dopamine, Ascorbic Acid, and Uric Acid

**DOI:** 10.3390/nano8010017

**Published:** 2017-12-30

**Authors:** Chang-Seuk Lee, Su Hwan Yu, Tae Hyun Kim

**Affiliations:** Department of Chemistry, Soonchunhyang University, Asan 31538, Korea; eriklee0329@sch.ac.kr (C.-S.L.); shsh422@naver.com (S.H.Y.)

**Keywords:** electrochemically reduced graphene oxide (ERGO), gold nanoparticles (AuNPs), hybrid nanocomposites, dopamine, ascorbic acid, uric acid

## Abstract

Here, we introduce the preparation of the hybrid nanocomposite-modified electrode consisting of reduced graphene oxide (RGO) and gold nanoparticles (AuNPs) using the one-step electrochemical method, allowing for the simultaneous and individual detection of dopamine (DA), ascorbic acid (AA), and uric acid (UA). RGO/AuNPs nanocomposite was formed on a glassy carbon electrode by the co-reduction of GO and Au^3+^ using the potentiodynamic method. The RGO/AuNPs nanocomposite-modified electrode was produced by subjecting a mixed solution of GO and Au^3+^ to cyclic sweeping from −1.5 V to 0.8 V (vs. Ag/AgCl) at a scan rate 10 mV/s for 3 cycles. The modified electrode was characterized by scanning electron microscopy, Raman spectroscopy, contact angle measurement, electrochemical impedance spectroscopy, and cyclic voltammetry. Voltammetry results confirm that the RGO/AuNPs nanocomposite-modified electrode has high catalytic activity and good resolution for the detection of DA, AA, and UA. The RGO/AuNPs nanocomposite-modified electrode exhibits stable amperometric responses for DA, AA, and UA, respectively, and its detection limits were estimated to be 0.14, 9.5, and 25 μM. The modified electrode shows high selectivity towards the determination of DA, AA, or UA in the presence of potentially active bioelements. In addition, the resulting sensor exhibits many advantages such as fast amperometric response, excellent operational stability, and appropriate practicality.

## 1. Introduction

Dopamine (DA) plays a key role in the central nervous, renal, and hormonal systems of human bodies [1]. Abnormal levels of DA are diagnostic factors of several diseases such as schizophrenia [2], Parkinson’s disease [3], Alzheimer’s disease [4], and Huntington’s disease [5]. Therefore, a highly sensitive determination of DA levels is needed in the early diagnosis of neurological disorders. Many researchers have proposed different methods for detection of DA, including fluorescence [6,7], surface enhanced Raman scattering [8], chromatography [9], microdialysis [10,11], and electrochemical methods [12,13,14,15]. Among them, electrochemical methods have a lot of advantages such as simplicity, low cost, short time of operation, high sensitivity, and availability of in-situ monitoring. However, electrochemical detection of DA can be disturbed by other biological molecules such as ascorbic acid (AA), and uric acid(UA) [16]. The high levels of AA can overwhelm the electrochemical signal of DA. Moreover, the voltammetric response of UA is similar to that of DA, making it difficult to detect DA selectively. To overcome this limitation, several approaches have been developed using the modified electrodes based on catalytic nanomaterials. Combination of various nanomaterials such as nanoparticles [17], bimetallic nanocomposites [18], ionic liquids [19], polymers [20], MoS_2_ [21], and graphene [22,23,24,25,26,27,28,29,30,31] have been applied to modify the electrodes for DA determination.

Among those nanomaterials, graphene has been extensively used as a material for modifying electrodes, owing to its unique structural and electronic properties [22,23]. Considering the characteristic of the graphene and its inherent electrocatalytic property, modified electrodes with graphene-related nanomaterials such as pristine graphene [24], chemically reduced graphene oxide (CRGO) [25,26,27], and electrochemically reduced graphene oxide (ERGO) [28,29,30] have been reported to effectively detect DA, AA, and UA. On the other hand, the modified electrodes of metal nanoparticles (NPs) have received foremost interest in electroanalysis due to good biocompatibility, large surface area, and excellent catalytic property [31]. Besides, graphene decorated with metal NPs has become increasingly important, because the graphene-metal hybrid nanocomposites can show the synergic effect of electrocatalytic behavior of both graphene and metal NPs. In particular, RGO can provide a versatile scaffold for NPs to form hybrid nanocomposite with improved properties, owing to its defects and oxygen functional groups (–OH, C=O, –COOH). In recent times, many types of graphene-metal hybrid nanocomposites [20,21] have been suggested for the modification materials of the electrochemical biosensor. The nanocomposite-modified electrodes are usually prepared using RGO, which is, however, obtained mostly by chemical reduction with toxic reducing agents. This can cause problems for human health and the environment. Moreover, electrode modifications with RGO are commonly achieved using the drop-casting method [32,33], which can cause variations in film thickness or internal structure, due to differences in evaporation rates across the substrate or concentration fluctuations. Furthermore, the preparation to composite RGO with NPs may require multiple and time-consuming steps of preparation or more sensitive handling. Therefore, the eco-friendly and simple preparation of graphene-based hybrid nanocomposites is very necessary.

**Scheme 1:**
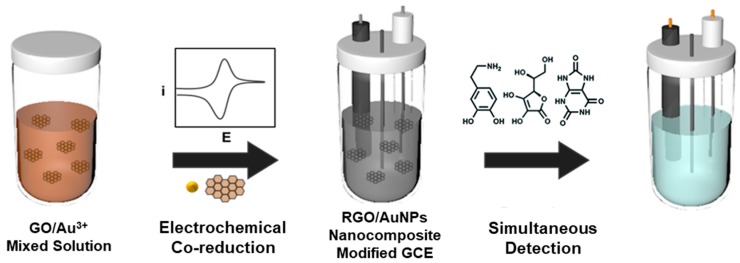
Fabrication of RGO/AuNPs nanocomposite modified electrode via one-step electrochemical co-reduction for simultaneous detection of DA, AA, and UA.

In this study, we report a simple electrochemical fabrication of RGO/AuNPs nanocomposite modified electrode by one-step electrochemical co-reduction of RGO and Au^3+^. It is worth noting that AuNPs were utilized here to provide a highly effective surface area and better mass transport of target analytes to the electrocatalyst [34,35]. The as-prepared electrode was used for the simultaneous and individual detection of DA, AA, and UA. Scheme 1 shows the overall process for fabricating the RGO/AuNPs nanocomposite modified electrode; as can be seen, the fabrication was performed in a mixed solution of GO and Au^3+^ using voltammetric cycling, which is a simple, fast, and eco-friendly process. Notably, this is one-step fabrication method via co-electrodeposition through simple voltammetric scanning exists without the need for any other processes, such as drop-casting and drying. The resulting electrode exhibited good electrocatalytic behavior, allowing for good sensitivity and selectivity with respect to individual and simultaneous determination of DA, AA, and UA without requiring any additional treatments. 

## 2. Results and Discussion

### 2.1. Preparation and Structure Characterization of RGO/AuNPs Nanocomposite-Modified Electrode

**Figure 1:**
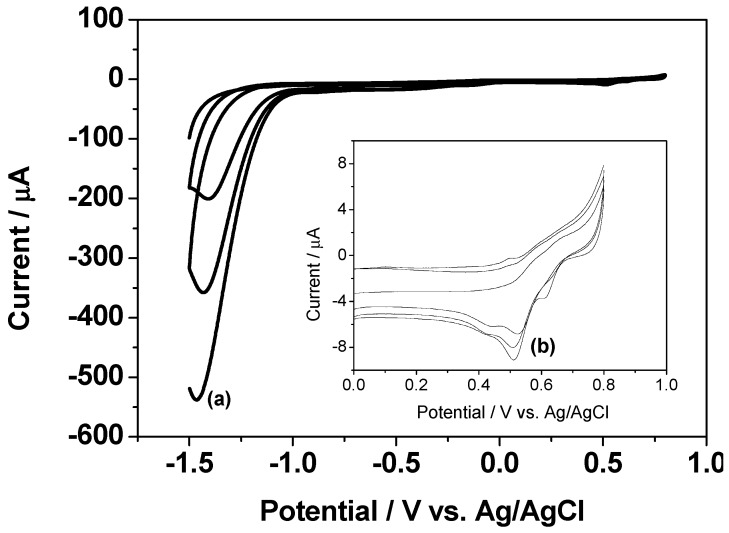
CV curve obtained on GCE in 10 mM PBS buffer solution (pH 7) containing 0.3 mg/mL GO and 0.8 mM HAuCl_4_ at a scan rate of 10 mV/s.

Figure 1 shows the typical cyclic voltammetry (CV) curve recorded during the co-reduction of GO and Au^3+^ on glassy carbon electrode (GCE) in 10 mM PBS buffer solution (pH 7) containing 0.3 mg/mL of GO and 0.8 mM HAuCl_4_. There is a large reduction peak (a) at approximately −1.4 V due to the reduction of the oxygen functional groups of GO, which increases gradually after cycles, differently from previous electrochemical reduction of GO [36]. This can be explained by the deposition of AuNPs with higher conductivity on GO [37]. The reduction peak (b) at approximately 0.52 V is related to the reduction process of Au^3+^, which leads to the formation of AuNPs on electrode surface. These results indicate RGO/AuNPs nanocomposite was electrodeposited on GCE after the voltammetric cycling.

**Figure 2:**
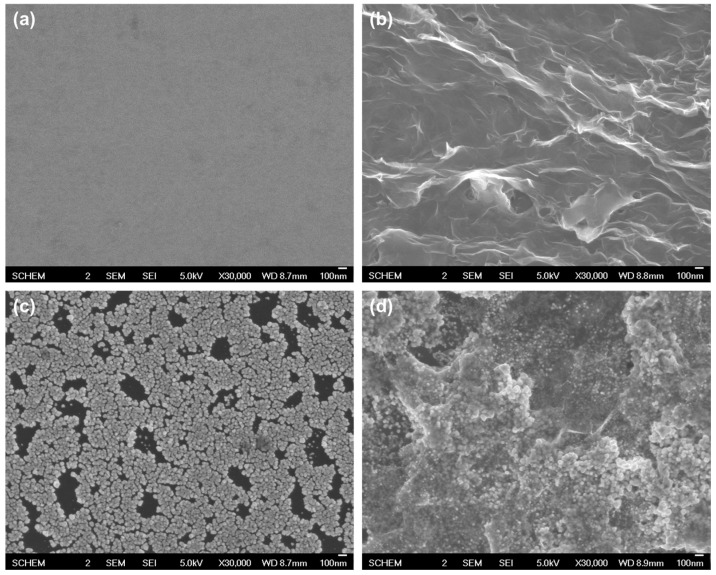
SEM images of (**a**) bare GCE, (**b**) RGO-GCE, (**c**) AuNPs-GCE, and (**d**) RGO/AuNPs-GCE.

The surface morphology of the as-prepared nanocomposite-modified electrode was characterized and compared using scanning electron microscopy (SEM) with those of RGO and AuNPs modified electrodes, prepared in same voltammetric condition with RGO/AuNPs nanocomposite. Figure 2 shows SEM images of (a) bare GCE, (b) RGO modified GCE (RGO-GCE), (c) AuNPs modified GCE (AuNPs-GCE), and (d) RGO/AuNPs nanocomposite modified GCE (RGO/AuNPs-GCE). The SEM image of GCE (a) displays relatively smooth surface, while the surface of RGO-GCE (b) is gauze-like shape with wrinkles, which provides large electroactive area on the electrode. The AuNPs-GCE (c) shows spherical-shaped and homogeneously distributed deposits with diameters of 38.7 ± 3.5 nm on the surface. The SEM image of RGO/AuNPs-GCE (d) shows that the AuNPs were densely and uniformly decorated along the surface of RGO. These results clearly confirm the formation of RGO/AuNPs nanocomposite on GCE.

**Figure 3:**
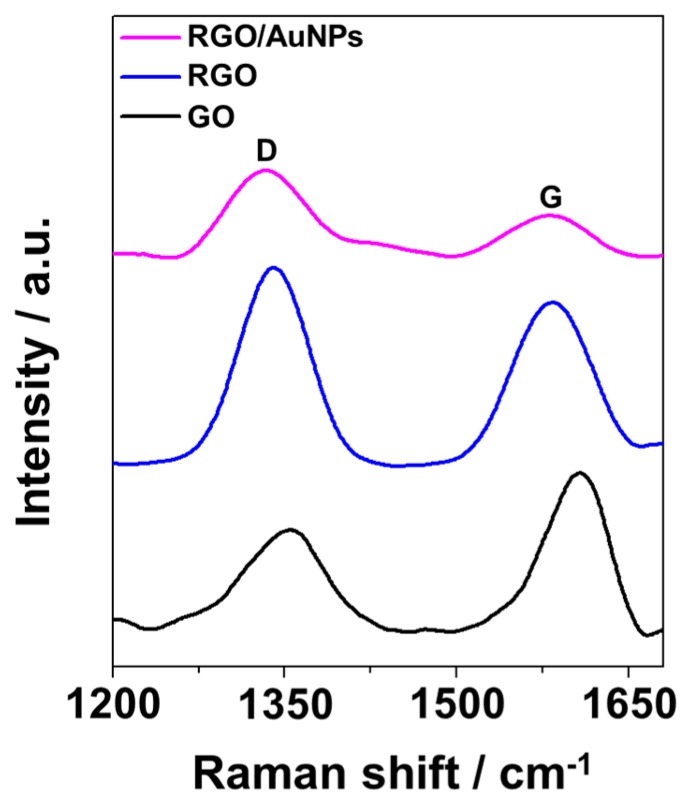
Raman spectra of GO, RGO, and RGO/AuNPs nanocomposites.

The structural property of RGO/AuNPs nanocomposite was investigated by Raman spectroscopy. Figure 3 shows the Raman spectra of GO, RGO, and RGO/AuNPs nanocomposite, respectively. The D band (~1354 cm^−1^) corresponds to the disorder in the sp^2^ carbon network, and the G band (~1607 cm^−1^) is associated with the tangential vibrations of the sp^2^ carbon atoms in the hexagonal planes [38]. The intensity ratio of the D and G bands (*I*_D_/*I*_G_) was employed to calculate the structural disorder; this ratio increased from 0.65 to 1.18 during the reduction of GO to RGO, suggesting a decrease in the average size of sp^2^ domains owing to the removal of the oxygen functional groups. *I*_D_/*I*_G_ (1.69) of the RGO/AuNPs is found to be higher, compared to that in GO, confirming the deoxygenation during the co-reduction of GO and Au^3+^. 

**Figure 4:**
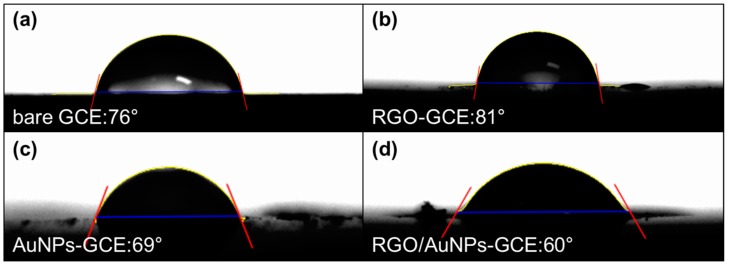
Contact angle analysis of (**a**) bare GCE, (**b**) RGO-GCE, (**c**) AuNPs-GCE, and (**d**) RGO/AuNPs-GCE.

To analyze the surface property of RGO/AuNPs nanocomposite, the changes in the wettability were determined through contact angle measurements. Figure 4 shows the wetting characteristics of bare GCE, RGO-GCE, AuNPs-GCE, and RGO/AuNPs-GCE, and their average equilibrium static contact angles are 76°, 80°, 69°, and 60°, respectively. GO is hydrophilic due to its oxygen containing functionalities, and bare GCE is slightly hydrophobic. However, after electrochemical reductive deposition of GO onto GCE, the contact angle of RGO-GCE shows more hydrophobic character than the bare GCE, which is attributed to the de-oxygenation or de-hydroxylation of GO. On the other hand, AuNPs-GCE and RGO/AuNPs-GCE show hydrophilic characters. This should be due to the gold surface oxidation during consecutive electro-oxidation and reduction cycling for the deposition of AuNPs [39]. The more hydrophilic property of RGO/AuNPs-GCE is attributed to the more immobilization of AuNPs on the wrinkled surface of RGO with large surface area.

### 2.2. Electrochemical Properties of RGO/AuNPs Nanocomposite-Modified Electrode

**Figure 5:**
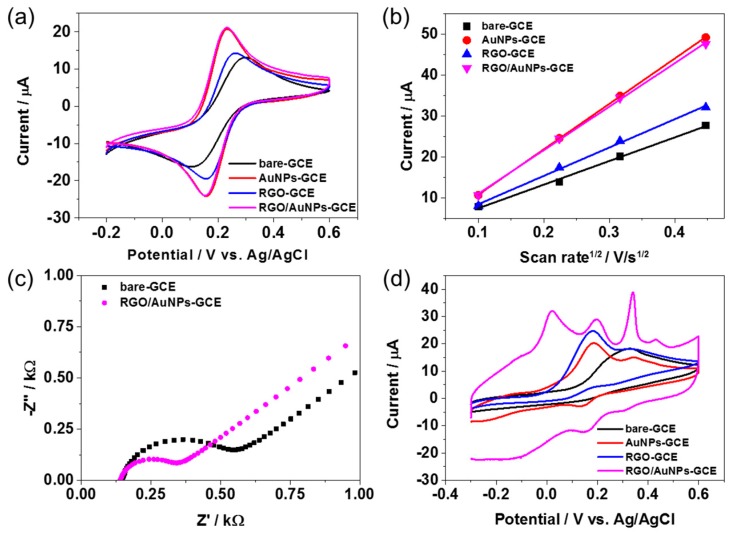
(**a**) CV curves of bare GCE, AuNPs-GCE, RGO-GCE, and RGO/AuNPs-GCE in 0.1 M KNO_3_ containing 2 mM [Fe(CN)_6_]^3−^ with 50 mV/s; (**b**) relationship between peak current and square root of scan rate for oxidation of bare GCE, AuNPs-GCE, RGO-GCE, and RGO/AuNPs-GCE in 0.1 M KNO_3_ containing 2 mM [Fe(CN)_6_]^3−^; (**c**) Nyquist plots of bare GCE, RGO/AuNPs-GCE in 0.1 M KCl containing 2 mM [Fe(CN)_6_]^3−^; (**d**) CV curves of bare GCE, AuNPs-GCE, RGO-GCE, and RGO/AuNPs-GCE in 0.1 M PBS buffer solution (pH 7.4) containing 100 μM UA, 1 mM AA, and 100 μM DA with 50 mV/s scan rate. Ag/AgCl electrode and Pt wire were introduced as reference and counter electrode, respectively.

CV and electrochemical impedance spectroscopy (EIS) measurements were carried out to analyze the electrochemical property of RGO/AuNPs-GCE using K_4_[Fe(CN)_6_] as the electrochemical probe. Figure 5a displays CV curves for bare GCE, AuNPs-GCE, RGO-GCE, and RGO/AuNPs-GCE in 0.1 M KNO_3_ solution containing 2 mM [Fe(CN)_6_]^3−^ at a scan rate of 50 mV/s, exhibiting a characteristic voltammetric response for [Fe(CN)_6_]^3−/4−^. However, the electron transfer reaction of [Fe(CN)_6_]^3−^ after the immobilization of AuNPs, RGO, or both on GCE was more facilitated with smaller peak separations of 70 mV, 87 mV, and 71 mV, respectively, compared to that in the bare GCE with 135 mV. In addition, AuNPs-GCE and RGO/AuNPs-GCE revealed significantly higher redox peak currents, indicating the better electrochemical performance. This should come from the electrocatalytic activity along with the large effective surface area of the modified electrodes. The effective surface areas of AuNPs-GCE, RGO-GCE, and RGO/AuNPs-GCE was 0.123 cm^2^, 0.083 cm^2^, and 0.121 cm^2^, respectively, as calculated using the Randles­Sevcik equation [40] from the CV curves obtained using 0.1 M KNO_3_ containing 2 mM [Fe(CN)_6_]^4−^ at different scan rates (Figure 5b). The capability of electron transfer of the RGO/AuNPs-GCE was further examined by EIS, as shown in Figure 5c. With respect to bare GCE, a decrease in the charge transfer resistance (*R*_ct_) by a factor of ~2 times was observed for the RGO/AuNPs-GCE, suggesting fast electron transfer occurred on the modified electrode surface. The electrocatalytic oxidations of DA, AA, and UA on the bare GCE, AuNPs-GCE, RGO-GCE, and RGO/AuNPs-GCE were primarily assessed by CV in 0.1 M PBS buffer solution (pH 7.4) containing 100 μM UA, 1 mM AA, and 100 μM DA at a scan rate of 50 mV/s. As shown in Figure 5d, in contrast with the bare GCE, AuNPs-GCE, and RGO-GCE, the RGO/AuNPs-GCE exhibited three well-resolved oxidation peaks corresponding to DA, AA, and UA. The ability of the RGO/AuNPs-GCE to promote the voltammetric resolution of DA, AA, and UA could be ascribed to the synergistic effect between RGO and AuNPs. The existence of oxide functional groups on the RGO would enable the nanocomposite to selectively interact with DA, AA, and UA via hydrogen bonds with the proton-donating group such as –NH and –OH [41,42]. In addition, π–π stacking interactions could induce facile electron transfer between RGO and target analytes [42]. Meanwhile, well-distributed AuNPs on the surface of RGO (Figure 2d) enhanced the catalytic activity of the nanocomposite by integrating AuNPs with RGO, which lead to the increases of electrode’s surface area and active sites for the oxidations of DA, AA, and UA [28]. These results evidenced that the RGO/AuNPs-GCE exhibits excellent electrocatalytic activity towards the oxidation of DA, AA, and UA. Thus, RGO/AuNPs-GCE could be used to efficiently discriminate between DA, AA, and UA, based on its voltammetric responses. 

**Figure 6:**
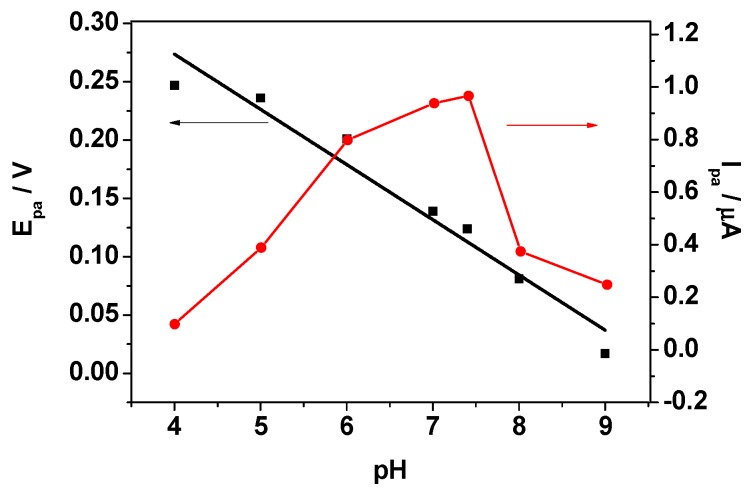
Effect of pH on the peak current and oxidation potential of DA at RGO/AuNPs-GCE.

Since the electroactivity of DA is pH dependent [43], the effect of pH on the oxidation process of DA was investigated by recording CV curves in various pH solutions (from 4.0 to 9.0) that contained 10 mM of DA. As shown in Figure 6, anodic peak current increased with an increase in pH up to 7.4, and then decreased at higher pH. Therefore, pH 7.4 was chosen for the rest of experiments to detect DA. In addition, the anodic peak potential (*E*_pa_) shifted to negative values with increasing pH. The relationship between the pH and oxidation potential was linear, and the related regression equation was found to be *E*_pa_ (V) = 0.463−0.047 pH (*R*^2^ = 0.95) for DA. The slope was 47 mV·pH^−1^, which is close to the theoretical value of 59 mV·pH^−1^ [44]. This result suggests that the oxidation of DA at RGO/AuNPs-GCE involves an equal number of protons and electrons [45], i.e., two protons and two electrons were involved in the oxidation process [46].

### 2.3. Selective Determination of DA, AA, and UA

**Figure 7:**
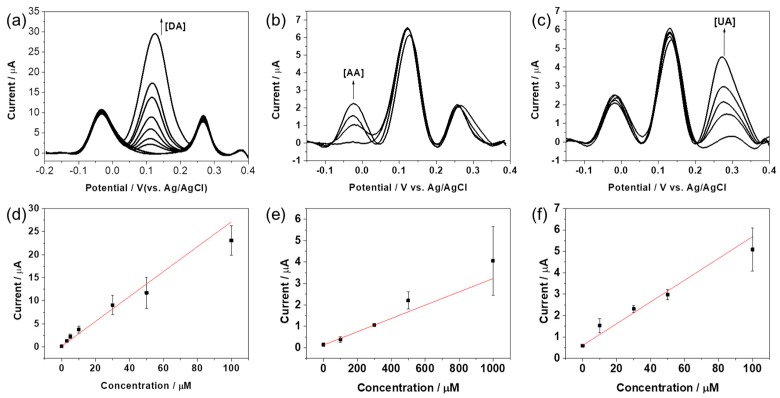
DPV curves of RGO/AuNPs-GCE in 0.1 M PBS (pH 7.4) mixed solution containing (**a**) 1 mM AA, 100 μM UA, and DA in different concentrations (0, 1, 3, 5, 10, 30, 50, 100 μM); (**b**) 100 μM DA, 100 μM UA, and AA in different concentrations (0, 100, 300, 500, 1000 μM); and (**c**) 1 mM AA, 100 μM DA, and UA in different concentrations (0, 10, 30, 50, 100 μM). Calibration curves of (**d**) DA, (**e**) AA, and (**f**) UA.

To further gain insight into detection and discrimination ability of RGO/AuNPs-GCE, differential pulse voltammetry (DPV) was performed in a 0.1 M PBS (pH 7.4) solution containing different concentration of DA, AA, and UA. As seen in Figure 7, the DPV curved towards DA, AA, or UA, when the increasing concentrations of the target molecule were added to the mixed solution of the other two analytes at constant concentrations. It can be seen that the oxidation waves of the three compounds are well separated and their current intensities rise with the increase of species’ concentration. As shown in Figure 7a,d, the oxidation peak currents of DA linearly increases with increasing concentrations of DA in the range from 0.1 to 100 μM with limit of detection (LOD) of 0.69 μM (*S*/*N* = 3). Notably, the changes of DA concentration have negligible influence on the oxidation behaviors of AA and UA. In a similar way, the oxidation peak currents of AA (Figure 7b,e) or UA (Figure 7c,f) increased linearly with increasing concentration in the range from 0.01 to 1 mM and 0.1 to 100 μM with LODs of 5.7 μM and 2.2 μM, respectively. These results confirm that the electrode exhibits simultaneous detection of DA, AA, and UA, as the oxidation waves of these analytes are well resolved.

### 2.4. Amperometric Responses of DA, AA, and UA

**Figure 8:**
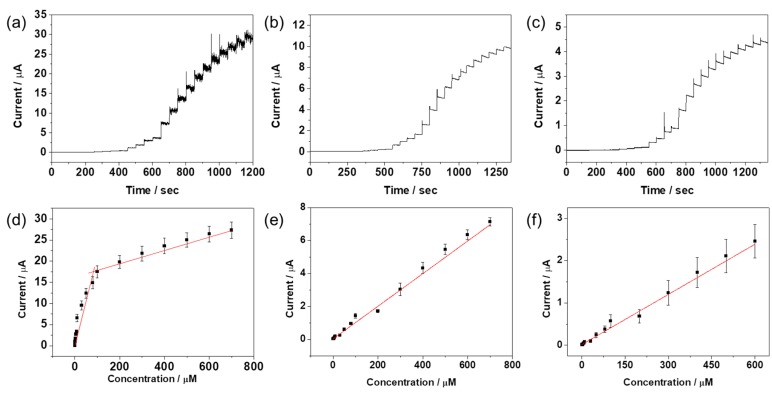
Amperometric responses of RGO/AuNPs-GCE to successive addition of (**a**) DA, (**b**) AA, and (**c**) UA in 0.1 M PBS (pH 7.4) under stirring. Working potentials were 0.2, −0.02, 0.265 V (vs. Ag/AgCl reference electrode) for DA, AA, and UA, respectively. Calibration plot of steady-state currents obtained at the RGO/AuNPs-GCE against concentrations of (**d**) DA, (**e**) AA, and (**f**) UA.

To evaluate the analytical feasibility of RGO/AuNPs-GCE, chronoamperometric measurements were performed in 0.1 M PBS (pH 7.4) at fixed potentials under constant stirring. A stock solution of the target analyte was added into the stirred 10 mL of 0.1 M PBS (pH 7.4) at 50 s intervals, and the final concentration and composition of the solution were adjusted to the desired value. The amperometric response of the RGO/AuNPs-GCE was monitored before and after the addition of the target analyte at selected working potential (0.2, −0.02, and 0.265 V for DA, AA, and UA, respectively). The amperometric current-time plots of DA, AA, and UA at the RGO/AuNPs-GCE are displayed in Figure 8a–c. The current signal rose rapidly with each addition of the target analyte, then reaching a steady-state current within 2 s, indicating a fast oxidation response behavior. The RGO/AuNPs-GCE show linear responses towards the oxidation of DA, AA, and UA. Their corresponding calibration curves are shown in Figure 8d–f; the relationship between the concentration of analyte and its amperometric currents are linear. Sensing performance analyses from amperometric and DPV methods are summarized in Table 1.

It is worth comparing the proposed modified electrode with other similar electrodes for DA determination [24,25,26,27,28,29,30]. Table 2 summarizes some characteristics of the proposed electrode and other modified electrodes, including LOD, detectable range, advantages, and disadvantages. Although some of the modified electrodes have some advantages, the proposed electrode has made some improvements in terms of analytical performance such as having a good linear range and low LOD. Most notably, our fabrication method is based on a one-pot simultaneous reduction of GO and gold precursor without any other processes such as drop-casting and drying, and is more facile and less time consuming than other methods.

### 2.5. Interference Study

**Figure 9:**
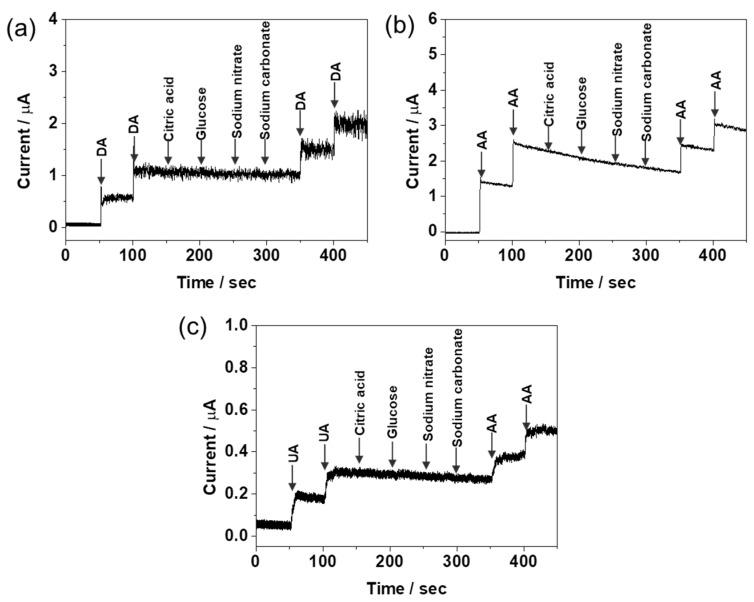
Amperometric responses of RGO/AuNPs-GCE upon successive addition of (**a**) 10 μM DA, (**b**) 100 μM AA, (**c**) 10 μM UA, and other chemicals of 10 μM to 0.1 M PBS buffer (pH 7.4). The applied potentials for AA, DA, and UA are −0.02, 0.2, and 0.265 V, respectively.

In the development of electrochemical biosensor, the study of selectivity by interfering species is very important factor. Various foreign species were tested to examine whether they would interfere with the detection of DA, AA, and UA. The RGO/AuNPs-GCE was dipped in a stirring solution and its amperometric response was monitored in the presence of 10 μM DA, 100 μM AA, and 10 μM UA with different interferents such as citric acid, glucose, sodium nitrate, and sodium carbonate (Figure 9). The applied potentials were −0.02, 0.2, and 0.265 V for DA, AA, and UA, respectively. Noticeably, no significant change of the response current was found in the presence of interferent. This clearly demonstrates that RGO/AuNPs-GCE performs with an excellent selectivity and little interference.

## 3. Materials and Methods

### 3.1. Materials

Graphite powder, dopamine (DA) hydrochloride, L-(+)-ascorbic acid (AA), uric acid (UA), citric acid (CA), glucose, potassium hexacyanoferrate (K_3_[Fe(CN)_6_]), phosphate buffer saline (PBS, pH 7.4, 0.1 M), and hydrogen tetrachloroaurate (III) (HAuCl_4_) were purchased from Sigma-Aldrich Co. (St. Louis, MO, USA). All the other chemicals used such as sodium hydroxide (NaOH), sodium nitrate (NaNO_3_), sodium carbonate (Na_2_CO_3_), potassium permanganate (KMnO_4_), hydrogen peroxide (H_2_O_2_), and sulfuric acid (H_2_SO_4_) were obtained from Duksan Pure Chemicals Co. (Ansan, Korea). All the reagents were analytical grade and were used without further purification. Deionized (DI) water was used throughout the work.

### 3.2. Instruments

The electrochemical measurements were performed with a CHI 660D electrochemical workstation (CH Instruments, Inc., Austin, TX, USA). A conventional three-electrode cell was used at room temperature, including glassy carbon electrode (GCE) as working electrode, Pt wire as counter electrode, and Ag/AgCl electrode as reference electrode, respectively. Electrochemical impedance spectroscopy (EIS) was performed in 2.0 mM K_3_Fe(CN)_6_ with 0.1 M KCl as supporting electrolyte. The morphology of the modified electrode was examined using scanning electron microscopy (SEM) system (JEOL, JSM 5600 LV, Tokyo, Japan). Contact angle images were taken on a PHOENIX-MINI (SEO Co. Ltd., Suwon, Korea) system. Raman spectroscopy was performed using a EnSpectr R532 Raman spectrometer (Enhanced Spectrometry, Inc., Torrance, CA, USA). 

### 3.3. Preparation of RGO/AuNPs Nanocomposite Electrode

Graphene oxide (GO) was synthesized using graphite powders by the modified Hummers method [47,48]. The synthesized GO was dispersed as 1.0 mg/mL in DI water, and the solution was ultrasonicated for 1 h. Before the co-reduction of GO and Au^3+^ ion, GCE was polished with 1.0, 0.3, and 0.05 μm alumina powder and then sonicated for 5 min in ethanol and DI water successively. After that, the GCE was electrochemically polished in 0.25 mM H_2_SO_4_ solution with CV; potential was scanned from −1.0 V to 1.0 V with 50 mV/s scan rate for 20 cycles. The GCE was immersed into a 10 mM of PBS buffer solution (pH 7.4) containing 0.3 mg/mL of GO and HAuCl_4_. The co-reduction of GO and Au^3+^ ion was performed with CV from −1.5 V to 0.8 V (vs. Ag/AgCl) at a scan rate 10 mV/s for 3 cycles.

### 3.4. Electrochemical Measurements

A 0.1 M PBS (pH 7.4) was used as the supporting electrolyte for electrochemical determination of DA, AA and UA, respectively. Before the measurement, the solution was deoxygenated with pure N_2_ gas for 10 min. 

## 4. Conclusions

We proposed one-step electrochemical preparation of RGO and AuNPs nanohybrid on a GCE by CV in 10 mM PBS buffer (pH 7) containing 0.3 mg/mL of GO and 0.8 mM HAuCl_4_. The analytical feasibility of the RGO/AuNPs-GCE was investigated by using CV, DPV, and amperometric measurements. The RGO/AuNPs-GCE exhibited good catalytic activity toward AA, DA, and UA oxidation, displaying the well-resolved potential peak separation and enhanced peak currents for the oxidation of the three analytes. The proposed electrode also showed excellent electrochemical sensing performance such as low LOD, wide linear range, fast response time, as well as good selectivity and sensitivity, which indicates the RGO/AuNPs can be a potential candidate for detection of AA, DA, and UA. The fabrication of the proposed electrode is simple and efficient that may contribute to develop a high performance electrochemical sensor.

## Figures and Tables

**Table 1:** Comparison of limits of detection (LOD) and sensitivity values with respect to DA, AA, and UA as determined by amperometric and DPV analysis.

Targets		DA	AA	UA
**Amperometry**	Linear regression equation	i_p_ (μA) = 7.6 × 10^−8^ + 0.43C (μM) *R*^2^ = 0.954 (0.14–100 μM), i_p_ (μA) = 1.4 × 10^−8^ + 0.023C (μM) *R*^2^ = 0.968 (100–700 μM)	i_p_ (μA) = 4.8 × 10^−8^ + 0.0081 C (μM) *R*^2^ = 0.988	i_p_ (μA) = 2.1 × 10^−8^ + 0.0035 C (μM) *R*^2^ = 0.979
	LOD (μM)	0.14	9.5	25
	Sensitivity (μA·μM^−1^)	0.43	8.1 × 10^−3^	3.5 × 10^−3^
**DPV**	Linear regression equation	i_p_ (μA) = 1.8 × 10^−7^ + 0.27 C (μM) *R*^2^ = 0.935	i_p_ (μA) = 1.2 × 10^−7^ + 0.0031 C (μM) *R*^2^ = 0.989	i_p_ (μA) = 6.0 × 10^−7^ + 0.051 C (μM) *R*^2^ = 0.977
	LOD (μM)	0.69	5.7	2.2
	Sensitivity (μA·μM^−1^)	0.27	3.1 × 10^−3^	0.051

**Table 2:** Comparison of various graphene-based, composite-modified electrodes towards the detection of dopamine.

Electrode Material	LOD (μM)	Sensitivity (μA/μM)	Range (μM)	Remarks	Reference
RGO/Au nanoplate	1.4	1.0	6.8–41	Multi-step, ERGO, Drop-casting	[28]
RGO-AuNPs-CSHMs	0.3	0.048	1–200	Multi-step, CRGO, Drop-casting	[25]
AgNPs/rGO	5.4	0.39	10–800	Multi-step, CRGO, Drop-casting	[26]
ERGO	0.5	0.482	0.5–60	Two-step, ERGO, Drop-casting	[29]
Pt/RGO	0.45	0.0391	10–170	Two-step, CRGO, Drop-casting	[27]
MgO/Gr/Ta	0.15	1.191	0.1–7	Multi-step, CVD-Graphene	[24]
ERGO/CFE	0.77	0.1024	1.5–224.82	One-step, ERGO	[30]
RGO/AuNPs	0.137	0.43	0.14–700	One-step, ERGO	This work
